# Modeling diffuse midline glioma through triple-electrode *in utero* electroporation of the developing mouse pons

**DOI:** 10.1371/journal.pone.0351079

**Published:** 2026-06-17

**Authors:** Madisen S. Mason, Hosbaldo Morales Murillo, Madisyn G. Dudek, Samantha E. Maher, Santos J. Franco

**Affiliations:** 1 Neuroscience Graduate Program, University of Colorado Anschutz, Aurora, Colorado, United States of America; 2 Summer Research Training Program, University of Colorado Anschutz, Aurora, Colorado, United States of America; 3 Science Research Seminar, Monarch High School, Boulder Valley School District, Louisville, Colorado, United States of America; 4 Department of Pediatrics, Section of Developmental Biology, University of Colorado Anschutz, Aurora, Colorado, United States of America; 5 Program in Pediatric Stem Cell Biology, Children’s Hospital Colorado, Aurora, Colorado, United States of America; University of North Carolina at Charlotte, UNITED STATES OF AMERICA

## Abstract

The leading cause of brain cancer–related death in children is diffuse midline glioma (DMG). A particularly aggressive DMG subtype is pontine DMG (formerly diffuse intrinsic pontine glioma, DIPG), which is caused by the histone mutation H3.3K27M. Because of its diffuse growth and location in a critical brainstem structure, therapeutic options are limited, and pontine DMG is considered universally fatal. The lack of appropriate animal models has hindered our understanding of the developmental origins and progression of pontine DMG, which in turn has limited the development of effective therapeutics. To address this barrier, several labs have developed mouse *in utero* electroporation approaches to express canonical DMG mutant oncogenes in the developing pons. In this manuscript and accompanying protocol, we describe a modified *in utero* electroporation strategy to generate DMG tumors in the developing mouse pons. Our protocol incorporates a single plasmid construct that expresses canonical DMG oncogenes, eliminating the need to co-electroporate multiple plasmids. We also employ a triple-electrode configuration to precisely target neural progenitors lining the fourth ventricle, which give rise to cells in the pons. As the embryos continue to develop *in utero* and postnatally, they form large, diffuse brainstem tumors with molecular characteristics of pediatric pontine DMG, allowing us to model the formation and progression of this deadly pediatric brain cancer.

## Introduction

Tumors of the central nervous system are the most common cancer-related cause of death in children. Pediatric high-grade gliomas are particularly aggressive and deadly, accounting for nearly half of all deaths caused by pediatric brain tumors [1]. Importantly, high-grade gliomas in children differ significantly from those that develop in adulthood in terms of their cellular origins and biological drivers [2,3]. These differences are reflected in the fact that clinical trials and treatments based on adult high-grade gliomas have shown no significant improvement in clinical outcomes in children [4]. Tumors also arise in a specific spatiotemporal pattern, typically in middle childhood [5], suggesting that tumor cells arise from dysregulation of a normal developmental process. Because of its location, current treatment options are limited, and prognosis is poor [6].

In recent years, significant advancements in patient-derived cell lines and animal models have provided a solid foundation to expand our understanding of the molecular underpinnings of these tumors. Gain-of-function mutations in genes encoding histone 3 (H3) variants H3.1 and H3.3 are found in a substantial proportion of pediatric high-grade gliomas, and these mutations are always present in diffuse midline glioma (DMG, H3K27-altered) [7]. Mutation of lysine 27 to methionine on H3 (H3K27M mutations) causes loss of trimethylation at this position, resulting in aberrant epigenetic regulation in affected cells [8,9]. The resulting transcriptional changes can significantly impact neural progenitor cells that rely on precise gene regulatory networks to produce the correct types of cells in proper numbers. H3K27M mutations in these tumors are often combined with genetic loss of *TP53* and amplification of *PDGFRA* [2,3,10–12]. Together, these studies indicate that the primary driver of DMG is epigenetic and transcriptional dysregulation in neural progenitors and glial precursors, combined with suppression of apoptosis and activation of growth factor pathways.

Most current animal models of DMG involve orthotopic xenografts of human tumor cells into adult immunodeficient mice. Though these models produce tumors, they require immunodeficient mice to generate tumors, and the xenografted tumors grow in adult mice, not in the developing brain as in human patients. A few genetically engineered mouse models of DMG have been developed to generate tumors that form in early embryonic and postnatal development in animals with competent immune systems [13–19]. However, studies using genetically engineered mice have been relatively sparse, possibly because of the challenges of engineering multiple inducible mutant alleles into the same mouse line. Recently, *in utero* electroporation (IUE) has been used to generate spatiotemporally defined gliomas with specific genetic drivers for pediatric DMG in an immunocompetent setting [20,21]. This strategy overcomes many of the limitations of prior approaches and has been used in several studies since these initial reports [22–28]. IUE has proven to be an invaluable tool for the study of brain development, and our group has utilized this approach extensively [29–32].

Here we describe an adaptation of the previously published *in utero* electroporation technique that employs a single plasmid encoding multiple oncogenic drivers and a triple-electrode configuration to study pontine DMG (previously known as diffuse intrinsic pontine glioma, DIPG). Previous protocols have already described the materials and general steps of IUE of the cerebral cortex [33,34], so we do not readdress those details here. Our modified setup incorporates a third electrode in addition to standard forceps-type electrodes, which allows more precise targeting of the developing pons. By adjusting the position and polarity of the electrodes, neural progenitors in the embryonic pons primordium can be electroporated, resulting in spatially specific tumor formation in the mouse developing pons.

## Materials and methods

The protocol described in this peer-reviewed article is published with full details on protocols.io [35], (**dx.doi.org/10.17504/protocols.io.5jyl8dy7dg2w/v1**) and is included for printing as (S1 File) with this article.

### Experimental animals

The following mouse lines were obtained from The Jackson Laboratory: B6 (C57BL/6J, stock no. 000664); 129 (129x1/SvJ, stock no. 000691). We also obtained timed pregnant Crl:CD1(ICR) mice (strain no. 022) from Charles River, which arrived at embryonic day (E) 12.5 and on which we performed electroporations at E15.5. For timed matings of 129;B6 hybrid mice, we considered embryos to be at gestation day 0.5 on the day when the vaginal plug was detected.

Experiments involving animals were performed according to the guidelines from the Institutional Animal Care and Use Committee of the University of Colorado–Anschutz Medical Campus and were approved in CU-AMC IACUC protocol #19. All mice were maintained according to the Guide for the Care and Use of Laboratory Animals.

For *in utero* electroporation surgeries, we used the following pre-analgesia and anesthesia protocol: The pregnant dam was anesthetized following UC-Denver Veterinary Formulary for isoflurane, delivered via a nose cone and inhaled by the dam. The concentration of isoflurane was administered to effect by adjusting the percent of displacement of O_2_ with a precision vaporizer and compressed O_2_. Induction of anesthesia was achieved with <2 min exposure to 3–5% Isoflurane. Maintenance anesthesia was between 1.5–2.5% Isoflurane. Pre-analgesia was administered before surgery began (Meloxicam, 1–2 mg/kg, 50–100 ul by subQ injection). Following surgery, we minimized pain and distress by administering Meloxicam 1–2 times daily for up to 3 days.

When any animal developed signs of severe pain, discomfort, or infection as a result of surgeries or tumor growth, the veterinary staff was consulted and the animal euthanized if necessary. Mice were euthanized by CO_2_ asphyxiation followed by cervical dislocation, consistent with the recommendations from the American Veterinary Medical Association Guidelines for Euthanasia of Animals.

### Expression plasmids

PB-pCIG has been previously described [32]. TRIONCO plasmid was custom synthesized and purchased from GenScript Biotech. The plasmid comprises mouse *H3f3a* with a K27M mutation fused to GFP at the C-terminus (H3^K27M^-GFP), a P2A sequence, mouse *Trp53* with a dominant-negative R270H mutation fused to a V5 tag at the C-terminus (p53^R270H^-V5), a T2A sequence, and mouse *Pdgfra* with a constitutively-active D842V mutation (PDGFRA^D842V^), all cloned into the PB-pCIG backbone missing the IRES-GFP. The plasmid was confirmed by whole-plasmid DNA sequencing (plasmidsaurus). Full sequence available upon request. PB-DoubleUP was cloned by inserting a PCR fragment from Double UP sfGFP to mScarlet [36] into the PB-pCIG backbone missing the IRES-GFP using NEBuilder HiFi (New England Biolabs). CMV-mPB plasmid [29] expressing *piggyBac* transposase was co-electroporated to permit stable integration of the plasmids into electroporated progenitors.

### Immunohistochemistry and confocal imaging

**Fig 1:**
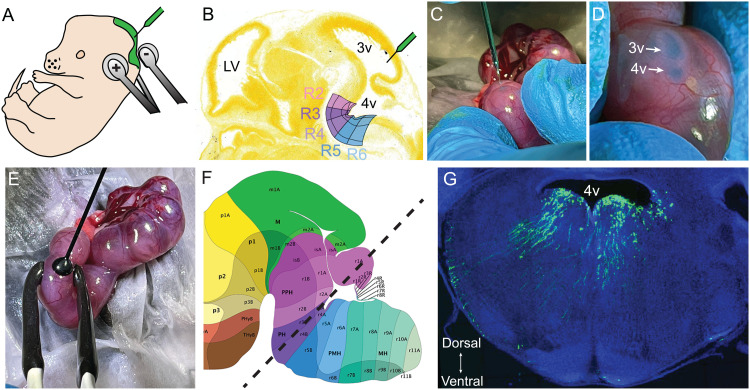
Targeting the embryonic pons by *in utero* electroporation using triple electrodes. **(A)** Schematic of IUE with injection into the 4^th^ ventricle and triple electrode placement. **(B)** Feulgen-HP yellow stain of a sagittal section of a mouse brain at E13.5, overlaid with anatomical annotations for target rhombomeres 2-6 (R2-6). From the Allen Reference Atlas – Developing Mouse Brain [brain atlas], available from atlas.brain-map.org/. **(C-E)** Images of an IUE procedure showing injection of DNA mix into an embryo using a glass microcapillary needle **(C)**, filling of the 3^rd^ and 4^th^ ventricles (arrows) with DNA mix, visualized by Fast Green dye **(D)**, and placement of triple electrodes around the injection sites **(E)**. **(F)** Atlas of a mouse brain at E17.5, showing anatomical annotations from the Allen Reference Atlas – Developing Mouse Brain [brain atlas], available from atlas.brain-map.org/. Dashed line shows oblique angle of sectioning for image in **(G)**. **(G)** Results of an IUE of control sfGFP at E15.5, dissected at E17.5 and cut obliquely as in **(F)**. Image shows electroporated neural progenitors with cell bodies in the ventricular zone of the 4^th^ ventricle and their radial processes spanning the entire pons. Abbreviations: LV, lateral ventricles; 3v, 3^rd^ ventricle; 4v, 4^th^ ventricle.

Embryonic brains were fixed in 4% paraformaldehyde (PFA) overnight at 4°C. Brains were sectioned obliquely (see Fig 1F) at 100 µm with a vibrating microtome. Free-floating sections were placed in 24-well plates and blocked with 500 μl of 10% donkey serum and 0.2% Triton X-100 in 1 × PBS for 2 h at RT. Blocking solution was then removed, and sections were incubated with primary antibodies in 10% donkey serum and 0.2% Triton X-100 in 1 × PBS overnight (16 h) at 4°C. Primary antibody solution was then removed, and sections were washed at RT with 1 × PBS three times for 5 min each. After washing, sections were incubated with secondary antibodies in 1 × PBS for 1 h at RT. Sections were then washed again using 1 × PBS three times for 5 min each. Sections were mounted on glass slides with ProLong Diamond Antifade Mountant (Thermo Fisher Scientific). Images were captured using a Zeiss LSM 900 laser scanning confocal microscope in Airyscan 2 Multiplex 4Y mode at 20× or 40 × magnification. Antibodies used for immunostaining are listed in **Biological and chemical reagents** table below. The concentration of each primary antibody used was the following: mouse anti-H3K27M (1:500), mouse anti-H3K27me3 (1:500), goat anti-OLIG2 (1:1000), rabbit anti-SOX2 (1:500), rat anti-PDGFRA (1:1000), chicken anti-GFP (1:500), and DAPI (1:10,000). Donkey secondary antibodies conjugated to Alexa Fluor 488, Rhodamine Red-X, or Alexa Fluor 647 were used at 1:500.

### Biological and chemical reagents

**Table:** 

REAGENT or RESOURCE	SOURCE	IDENTIFIER
Mouse strains		
B6 (C57BL/6J)	The Jackson Laboratory	stock no. 000664
129 (129X1/SvJ)	The Jackson Laboratory	stock no. 000691
Crl:CD1(ICR)	Charles River	strain no. 022
DNA plasmids		
CMV-mPB	Winkler et al., 2018	N/A
pPB-CAG-IRES-GFP (PB-pCIG)	Tran et al., 2023	N/A
TRIONCO	This paper	N/A
PB-DoubleUP	This paper	N/A
Double UP sfGFP to mScarlet	Gift from Erik Dent; Addgene	Cat# 120261; RRID: Addgene_120261
Antibodies		
Goat anti-OLIG2	R&D Systems	Cat# AF2418; RRID: AB_2157554
Rat anti-PDGFRα	Thermo Fisher Scientific	Cat# 14-1401-82, RRID: AB_467491
Rabbit anti-SOX2	Thermo Fisher Scientific	Cat# PA1–094; RRID: AB_2539862
Chicken anti-GFP	Thermo Fisher Scientific	Cat# A10262; RRID: AB_2534023
Mouse anti-V5 tag, DyLight 650	Thermo Fisher Scientific	Cat# MA5–15253-D65; RRID: AB_2537642
Rabbit anti-H3K27M	Cell Signaling	Cat# 74829; RRID: AB_2799861
Rabbit anti-H3k27me3	Epicypher Inc	Cat# 13–0055; RRID: AB_3665059
Donkey secondary antibody anti-Chicken Alexa Fluor 488	Jackson ImmunoResearch	Cat# 703-545-155; RRID: AB_2340375
Donkey secondary antibody anti-Goat Alexa Fluor 647	Jackson ImmunoResearch	Cat# 705-605-147; RRID: AB_2340437
Donkey secondary antibody anti-Rabbit Rhodamine Red-X	Jackson ImmunoResearch	Cat# 711-295-152; RRID: AB_2340613
Donkey secondary antibody anti-Rat Rhodamine Red-X	Jackson ImmunoResearch	Cat# 712-297-003; RRID: AB_2340679

## Results and discussion

### Triple electrode approach to electroporate 4^th^ ventricle progenitors *in utero*

Using the 4^th^ ventricle *in utero* electroporation protocol [35] (see also document S1 File for a printable protocol), we injected an electroporation mix containing a super folder GFP-expressing control vector (PB-DoubleUP) and *piggyBac* transposase (CMV-mPB) into the caudal end of the 3^rd^ ventricle (Fig 1A and Fig 1C) of embryos at embryonic day 15.5 (E15.5). Angling the injection needle caudally toward the 4^th^ ventricle allowed the plasmid mix to flow into the 4^th^ ventricle via the cerebral aqueduct (Fig 1A and Fig 1B). Proper injection into the 4^th^ ventricle was visualized by the Fast Green dye in the electroporation mix and identified as a small diamond-shaped spot caudal to the 3^rd^ ventricle, which was also often filled (Fig 1C). The ventricular zone along the 4th ventricle contains neural progenitors in rhombomeres 2–4 that give rise to the developing pons (Fig 1B). Using a triple electrode configuration [37], we electroporated the pons ventricular zone progenitors. We placed tweezer-style cathode electrodes on the lateral sides of the embryo’s upper neck on either side of and just ventral to the 4^th^ ventricle, and we placed the single anode electrode directly on the top of the 4^th^ ventricle (Fig 1E). This strategy allowed us to drive the electric current from the electroporator from dorsomedial to ventrolateral, thus introducing the plasmid DNA into progenitors in the ventricular zone of the 4^th^ ventricle. We dissected brains at E17.5 and cut oblique sections through the developing pons (Fig 1F). Confocal imaging revealed GFP-expressing neural progenitors in both the basal and alar plates of rhombomeres 2–4, with their cell bodies located in the ventricular zone and long radial processes spanning to the ventral surface of the pons (Fig 1G). Thus, this protocol allows efficient electroporation of neural progenitors throughout the ventricular zone of rhombomeres 2–4 that give rise to neurons, astrocytes, oligodendrocytes, and ependymal cells of the pons tegmentum [38].

Implementation of the triple-electrode configuration allowed us to improve targeting precision of neural progenitor cells lining the pons ventricular zone. This strategy can also electroporate progenitors in the lower rhombic lip if the tweezer electrodes are placed slightly more dorsally around the 4^th^ ventricle. The rhombic lip is a germinal matrix lining the dorsolateral edges of the 4^th^ ventricle, near the roofplate, that produces a highly diverse populations of cells in the brainstem and cerebellum [39]. The rhombic lip is separated into two distinct regions: the upper (rostral or cerebellar) rhombic lip in rhombomeres 1 and 2 that give rise to cells in the cerebellum [40], and the lower (caudal or hindbrain) rhombic lip in rhombomeres 2–7 that give rise to neurons that migrate tangentially to populate precerebellar nuclei, including pontine nuclei in the basilar pons [41]. Changing electrode placement or polarity can more effectively target the upper and/or lower rhombic lip if desired. We have also found that using just the tweezer-style electrodes alone, with one side as the cathode and the other the anode, can target either the left or right side of the rhombic lip depending on electrode placement.

### Validation of the TRIONCO Plasmid

**Fig 2:**
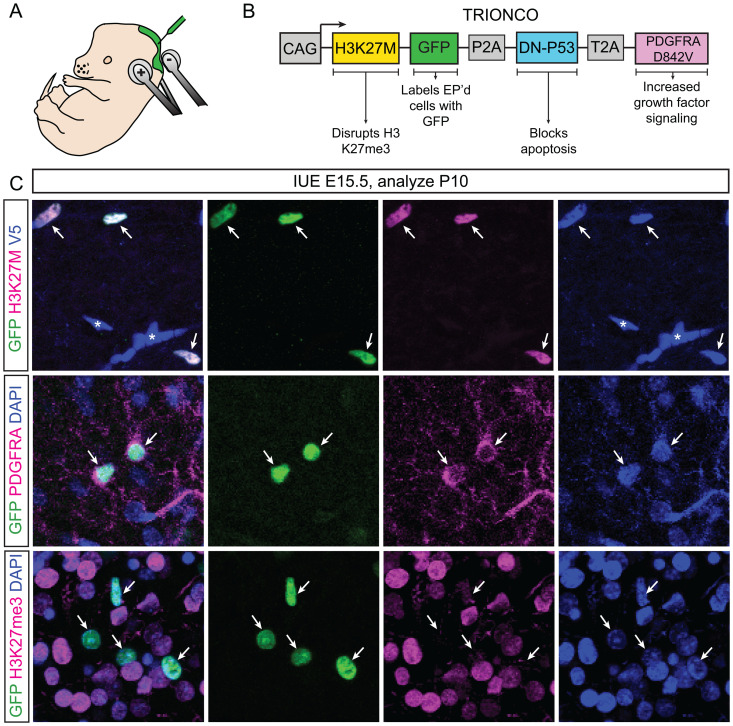
Validation of oncoprotein expression in brains electroporated with the TRIONCO plasmid. (**A)** Schematic of IUE with injection into the 4^th^ ventricle and triple electrode placement. **(B)** Schematic of TRIONCO plasmid to express DMG driver oncogenes, with descriptions of the functions of the encoded cDNAs. **(C)** Representative images of brains electroporated at E15.5 and analyzed at P10. Oblique sections were cut and stained for GFP (green), H3K27M (magenta), and V5 (blue) [top row], GFP (green), PDGFRA (magenta), and DAPI (blue) [middle row], or GFP (green), H3K27me3 (magenta), and DAPI (blue) [bottom row]. Merged channels shown in the left column, individual images at right. Arrows point at GFP+ cells that also co-express the other markers.

After demonstrating that we could target the pons ventricular zone, we next wanted to use this technique to generate pontine DMG tumors. Toward this goal, we first generated a single piggyBac plasmid (TRIONCO) encoding three canonical DMG oncogenes: 1) H3.3^K27M^, a point mutation that globally reduces histone methylation on lysine 27 (H3K27me3), 2) p53^R270H^, a dominant-negative version of p53 that blocks apoptosis, and 3) PDGFRA^D842V^, a constitutively-active version of platelet-derived growth factor receptor alpha (Fig 2A and Fig 2B). This combination of oncogenes has been shown previously to cause high-grade gliomas in a mouse *in* *utero* electroporation model [21]. However, in prior studies the oncogenes were encoded on separate plasmids and co-electroporated. Simultaneously electroporating multiple plasmids at similar molar ratios can result in relatively high co-expression rate [42,43]. However, we found in early pilot experiments using the co-electroporation approach that many cells were expressing only the constitutively active PDGFRA^D842V^, and that this could result in significant hyperplasia even in the absence of H3.3^K27M^ or p53^R270H^. Therefore, we encoded all 3 oncogenes on a single plasmid, referred to here as TRIONCO, to ensure co-expression in electroporated cells.

To validate our plasmid, we first electroporated TRIONCO at E15.5 and allowed the embryos to develop to postnatal day 10 (P10). We then dissected the electroporated brains and performed immunohistochemistry to verify expression of the encoded oncogenic proteins. Staining for antibodies against the mutant H3.3^K27M^ protein, the V5 tag fused to p53^R270H^, and PDGFRA demonstrated co-expression of all three proteins in GFP+ electroporated cells (Fig 2C). We also verified lower levels of H3K27me3 signal in GFP+ cells compared to neighboring cells that were not electroporated (Fig 2C), indicating that the H3K27M mutation was able to reduce H3K27me3 levels. Together, these data demonstrate proper expression of all 3 oncogenes after TRIONCO electroporation, leading to reduced histone 3 lysine 27 trimethylation levels.

### Generation of diffuse midline glioma tumors

**Fig 3:**
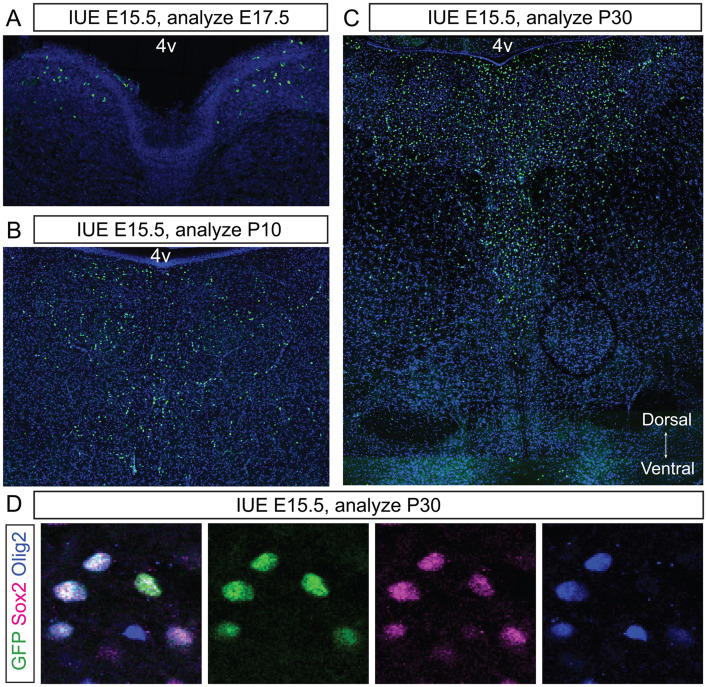
Mice electroporated with TRIONCO progressively develop diffuse tumors with molecular characteristics of pDMG. **(A-C)** Representative images of brains electroporated with TRIONCO at E15.5 and analyzed at various ages after IUE. At E17.5 (A) there are dozens of electroporated cells (green nuclei) per section. By P10 (B) electroporated cells have expanded into hundreds per section. At or P30 there are thousands of tumor cells per section, diffusely scattered throughout the pons. **(D)** Representative images of brains electroporated at E15.5 and analyzed at P30, showing co-expression of DMG markers in GFP+ electroporated cells. Oblique sections were cut and stained for GFP (green), SOX2 (magenta), and OLIG2 (blue). Merged channels shown in the left column, individual channels at right.

We next analyzed electroporated brains at different timepoints after TRIONCO electroporation at E15.5 to assess cellular expansion and tumor growth. Two days after electroporation, at E17.5, a small number of GFP+ electroporated cells were found bilaterally in and near the ventricular zone of the 4^th^ ventricle (Fig 3A). The numbers of GFP+ cells were similar in TRIONCO (Fig 3A) and control PB-DoubleUP (Fig 1G), indicating relatively low levels of proliferation of electroporated cells at this timepoint. By P10, that small number of GFP+ cells has increased to a several hundred cells that were distributed diffusely away from the ventricle, indicating substantial proliferation and migration into the developing pons. By P30 there was a substantial expansion of GFP+ electroporated cells numbering in the thousands (Fig 3C). The GFP+ cells were located diffusely throughout the dorsal-ventral and medial-lateral axes of the mature pons, with a higher concentration along the midline (Fig 3C). Immunohistochemistry for canonical DMG markers SOX2 and OLIG2 showed that nearly all GFP+ cells electroporated with TRIONCO were SOX2 + , with the majority of those also expressing OLIG2 (Fig 3D). Thus, cells electroporated with TRIONCO using the triple electrode protocol expand diffusely throughout the pons and express canonical DMG markers found in human tumors. Implementation of the single plasmid and triple-electrode approach described here provides a robust and reproducible method to model pontine DMG formation and progression *in vivo*.

### Additional considerations

We have successfully used this protocol to electroporate embryos between E12.5 and E15.5. Injection of the electroporation mix and placement of electrodes can be more challenging at E12.5-E13.5 compared to E14.5-E15.5, leading to lower rates of successfully electroporated brains, especially for less experienced surgeons. Birth of electroporated pups typically occurs on gestational day 19.0–19.5, so it is recommended to minimize disturbing the pregnant dam for about 1 week after surgery to facilitate best survival rates. In a typical experiment >80% of injected embryos will be born and nearly all surviving pups will develop pontine DMG tumors, although reproducibility of this protocol is highly dependent on experience and skill levels.

We have had success performing this protocol on both the CD1 outbred strain and a hybrid of 129;B6 inbred strains, each of which has some advantages and disadvantages. Females from both strains tend to be good mothers, which facilitates pup survival. CD1 mice have larger average litter sizes, which can increase numbers of electroporated mice but also increases variability, surgery time, and chances of complications. 129;B6 hybrids have smaller litter sizes than CD1, but more consistent developmental stages across all embryos in the litter. For both strains, including enrichment and enhanced diets can positively impact pup survival. We have not attempted this specific protocol on a pure C57BL6/J inbred strain, although our prior experience with electroporation of the lateral ventricles indicated that females of this strain do not care for their pups very well after *in utero* electroporation surgery and therefore survival of electroporated pups is extremely low.

To date we have not seen obvious differences in tumors formed in mice electroporated at E13.5 compared to E15.5. However, neural progenitors give rise to different subtypes of cells at different developmental timepoints, raising the possibility that timing of electroporation can impact cell of origin and therefore tumor characteristics. Thus, electroporation timing should be determined for each experimental paradigm depending on study goals. Likewise, the time of analysis will be highly study dependent. We have found that cell numbers continue to expand in TRIONCO tumors at least through P60, although we have not yet carefully analyzed tumor growth over time in a quantitative manner. In future studies it will be useful to incorporate a strategy to co-electroporate an additional plasmid encoding Luciferase, allowing tumor growth to be tracked longitudinally by *in vivo* bioluminescence imaging.

## Supporting information

S1 File Step-by-step protocol, also available on protocols.io. dx.doi.org/10.17504/protocols.io.5jyl8dy7dg2w/v1. (PDF)

## References

[1] Ostrom QT, de Blank PM, Kruchko C, Petersen CM, Liao P, Finlay JL, et al. Alex’s lemonade stand foundation infant and childhood primary brain and central nervous system tumors diagnosed in the United States in 2007–2011. Neuro-Oncology. 2014;16(suppl_10):x1–36. doi: 10.1093/neuonc/nou327 PMC4277295 25542864

[2] Zheng L, Gong J, Yu T, Zou Y, Zhang M, Nie L, et al. Diffuse midline gliomas with histone H3 K27M mutation in adults and children: A retrospective series of 164 cases. Am J Surg Pathol. 2022;46(6):863–71. doi: 10.1097/PAS.0000000000001897 35416795 PMC9093723

[3] Vuong HG, Ngo TNM, Le HT, Jea A, Hrachova M, Battiste J, et al. Prognostic implication of patient age in H3K27M-mutant midline gliomas. Front Oncol. 2022;12:858148. doi: 10.3389/fonc.2022.858148 35371982 PMC8971724

[4] Hargrave D, Bartels U, Bouffet E. Diffuse brainstem glioma in children: Critical review of clinical trials. Lancet Oncol. 2006;7(3):241–8. doi: 10.1016/S1470-2045(06)70615-5 16510333

[5] Vallero SG, Bertero L, Morana G, Sciortino P, Bertin D, Mussano A, et al. Pediatric diffuse midline glioma H3K27- altered: A complex clinical and biological landscape behind a neatly defined tumor type. Front Oncol. 2023;12:1082062. doi: 10.3389/fonc.2022.1082062 36727064 PMC9885151

[6] Di Ruscio V, Del Baldo G, Fabozzi F, Vinci M, Cacchione A, de Billy E, et al. Pediatric diffuse midline gliomas: An unfinished puzzle. Diagnostics. 2022;12:2064. doi: 10.3390/diagnostics12092064 36140466 PMC9497626

[7] Louis DN, Perry A, Wesseling P, Brat DJ, Cree IA, Figarella-Branger D, et al. The 2021 WHO Classification of Tumors of the Central Nervous System: A summary. Neuro Oncol. 2021;23(8):1231–51. doi: 10.1093/neuonc/noab106 34185076 PMC8328013

[8] Angelico G, Mazzucchelli M, Attanasio G, Tinnirello G, Farina J, Zanelli M, et al. H3K27me3 loss in central nervous system tumors: Diagnostic, prognostic, and therapeutic implications. Cancers (Basel). 2024;16(20):3451. doi: 10.3390/cancers16203451 39456545 PMC11506073

[9] Harutyunyan AS, Krug B, Chen H, Papillon-Cavanagh S, Zeinieh M, De Jay N, et al. H3K27M induces defective chromatin spread of PRC2-mediated repressive H3K27me2/me3 and is essential for glioma tumorigenesis. Nat Commun. 2019;10(1):1262. doi: 10.1038/s41467-019-09140-x 30890717 PMC6425035

[10] Mackay A, Burford A, Carvalho D, Izquierdo E, Fazal-Salom J, Taylor KR, et al. Integrated molecular meta-analysis of 1,000 pediatric high-grade and diffuse intrinsic pontine Glioma. Cancer Cell. 2017;32(4):520-537.e5. doi: 10.1016/j.ccell.2017.08.017 28966033 PMC5637314

[11] Filbin MG, Tirosh I, Hovestadt V, Shaw ML, Escalante LE, Mathewson ND, et al. Developmental and oncogenic programs in H3K27M gliomas dissected by single-cell RNA-seq. Science. 2018;360(6386):331–5. doi: 10.1126/science.aao4750 29674595 PMC5949869

[12] Paugh BS, Zhu X, Qu C, Endersby R, Diaz AK, Zhang J, et al. Novel oncogenic PDGFRA mutations in pediatric high-grade gliomas. Cancer Res. 2013;73(20):6219–29. doi: 10.1158/0008-5472.CAN-13-1491 23970477 PMC3800209

[13] Lewis PW, Müller MM, Koletsky MS, Cordero F, Lin S, Banaszynski LA, et al. Inhibition of PRC2 activity by a gain-of-function H3 mutation found in pediatric glioblastoma. Science. 2013;340(6134):857–61. doi: 10.1126/science.1232245 23539183 PMC3951439

[14] Tomita Y, Shimazu Y, Somasundaram A, Tanaka Y, Takata N, Ishi Y, et al. A novel mouse model of diffuse midline glioma initiated in neonatal oligodendrocyte progenitor cells highlights cell-of-origin dependent effects of H3K27M. Glia. 2022;70(9):1681–98. doi: 10.1002/glia.24189 35524725 PMC9546478

[15] Larson JD, Kasper LH, Paugh BS, Jin H, Wu G, Kwon C-H, et al. Histone H3.3 K27M accelerates spontaneous brainstem glioma and drives restricted changes in bivalent gene expression. Cancer Cell. 2019;35(1):140-155.e7. doi: 10.1016/j.ccell.2018.11.015 30595505 PMC6570409

[16] Fortin J, Tian R, Zarrabi I, Hill G, Williams E, Sanchez-Duffhues G, et al. Mutant ACVR1 arrests glial cell differentiation to drive tumorigenesis in pediatric gliomas. Cancer Cell. 2020;37(3):308-323.e12. doi: 10.1016/j.ccell.2020.02.002 32142668 PMC7105820

[17] Tian R, Wu A, Laugesen E, Mak T, Fortin J. DIPG-13. New immunocompetent genetically-engineered mouse models of pediatric-type diffuse high-grade gliomas reveal mechanisms of tumor initiation and progression. Neuro-Oncology. 2023;25(Supplement_1):i15–i15. doi: 10.1093/neuonc/noad073.060

[18] Hoeman CM, Cordero FJ, Hu G, Misuraca K, Romero MM, Cardona HJ, et al. ACVR1 R206H cooperates with H3.1K27M in promoting diffuse intrinsic pontine glioma pathogenesis. Nat Commun. 2019;10(1):1023. doi: 10.1038/s41467-019-08823-9 30833574 PMC6399349

[19] Becher OJ, Hambardzumyan D, Walker TR, Helmy K, Nazarian J, Albrecht S, et al. Preclinical evaluation of radiation and perifosine in a genetically and histologically accurate model of brainstem glioma. Cancer Res. 2010;70(6):2548–57. doi: 10.1158/0008-5472.CAN-09-2503 20197468 PMC3831613

[20] Pathania M, De Jay N, Maestro N, Harutyunyan AS, Nitarska J, Pahlavan P, et al. H3.3K27M Cooperates with Trp53 Loss and PDGFRA Gain in Mouse Embryonic Neural Progenitor Cells to Induce Invasive High-Grade Gliomas. Cancer Cell. 2017;32(5):684-700.e9. doi: 10.1016/j.ccell.2017.09.014 29107533 PMC5687892

[21] Patel SK, Hartley RM, Wei X, Furnish R, Escobar-Riquelme F, Bear H, et al. Generation of diffuse intrinsic pontine glioma mouse models by brainstem-targeted in utero electroporation. Neuro Oncol. 2020;22(3):381–92. doi: 10.1093/neuonc/noz197 31638150 PMC7442382

[22] Collot R, Ruiz-Moreno C, Honhoff C, van den Broek TJM, Wezenaar AKL, Kloosterman DJ, et al. IGSF11-VISTA is a critical and targetable immune checkpoint axis in diffuse midline glioma. Cancer Cell. 2026;44(3):641-657.e9. doi: 10.1016/j.ccell.2025.12.020 41576930 PMC12994606

[23] McNicholas M, De Cola A, Bashardanesh Z, Foss A, Lloyd CB, Hébert S, et al. A Compendium of Syngeneic, Transplantable Pediatric High-Grade Glioma Models Reveals Subtype-Specific Therapeutic Vulnerabilities. Cancer Discov. 2023;13(7):1592–615. doi: 10.1158/2159-8290.CD-23-0004 37011011 PMC10326601

[24] du Chatinier A, Meel MH, Das AI, Metselaar DS, Waranecki P, Bugiani M, et al. Generation of immunocompetent syngeneic allograft mouse models for pediatric diffuse midline glioma. Neurooncol Adv. 2022;4(1):vdac079. doi: 10.1093/noajnl/vdac079 35733514 PMC9210310

[25] De Cola A, McNicholas M, Foss A, Lloyd C, Hébert S, Faury D, et al. DIPG-35. Identifying driver-specific vulnerabilities in paediatric high grade glioma subtypes. Neuro-Oncology. 2023;25(Supplement_1):i20–1. doi: 10.1093/neuonc/noad073.082

[26] Miklja Z, Yadav VN, Cartaxo RT, Siada R, Thomas CC, Cummings JR, et al. Everolimus improves the efficacy of dasatinib in PDGFRα-driven glioma. J Clin Invest. 2020;130(10):5313–25. doi: 10.1172/JCI133310 32603316 PMC7524471

[27] Messinger D, Harris MK, Cummings JR, Thomas C, Yang T, Sweha SR, et al. Therapeutic targeting of prenatal pontine ID1 signaling in diffuse midline glioma. Neuro Oncol. 2023;25(1):54–67. doi: 10.1093/neuonc/noac141 35605606 PMC9825316

[28] Chen CCL, Deshmukh S, Jessa S, Hadjadj D, Lisi V, Andrade AF, et al. Histone H3.3G34-Mutant Interneuron Progenitors Co-opt PDGFRA for Gliomagenesis. Cell. 2020;183(6):1617-1633.e22. doi: 10.1016/j.cell.2020.11.012 33259802 PMC7791404

[29] Winkler CC, Yabut OR, Fregoso SP, Gomez HG, Dwyer BE, Pleasure SJ, et al. The dorsal wave of neocortical oligodendrogenesis begins embryonically and requires multiple sources of sonic hedgehog. J Neurosci. 2018;38(23):5237–50. doi: 10.1523/JNEUROSCI.3392-17.2018 29739868 PMC5990977

[30] Fregoso SP, Dwyer BE, Franco SJ. Lmx1a drives Cux2 expression in the cortical hem through activation of a conserved intronic enhancer. Development. 2019;146(5):dev170068. doi: 10.1242/dev.170068 30770393

[31] Gutierrez MA, Dwyer BE, Franco SJ. Csmd2 is a synaptic transmembrane protein that interacts with PSD-95 and is required for neuronal maturation. eNeuro. 2019;6(ENEURO.0434-18.2019). 10.1523/ENEURO.0434-18.2019 PMC6506821 31068362

[32] Tran LN, Loew SK, Franco SJ. Notch signaling plays a dual role in regulating the neuron-to-oligodendrocyte switch in the developing dorsal forebrain. J Neurosci. 2023. doi: 10.1523/JNEUROSCI.0144-23.2023 PMC10573779 37640551

[33] Martínez-Garay I, García-Moreno F, Vasistha N, Marques-Smith A, Molnár Z. In utero electroporation methods in the study of cerebral cortical development. Walker DW. Prenatal and postnatal determinants of development. New York, NY: Springer New York. 2016. 21–39. doi: 10.1007/978-1-4939-3014-2_2

[34] Meyer-Dilhet G, Courchet J. In utero cortical electroporation of plasmids in the mouse embryo. STAR Protocols. 2020;1: 100027. doi: 10.1016/j.xpro.2020.100027 PMC7357676 32685931

[35] Mason MS, Franco SJ. In uteroelectroporation of mouse pons via the 4th ventricle v1. 2025. doi: 10.17504/protocols.io.5jyl8dy7dg2w/v1

[36] Taylor RJ, Carrington J, Gerlach LR, Taylor KL, Richters KE, Dent EW. Double UP: A dual color, internally controlled platform for in utero knockdown or overexpression. Front Mol Neurosci. 2020;13:82. doi: 10.3389/fnmol.2020.00082 32508591 PMC7251070

[37] Szczurkowska J, Cwetsch AW, dal Maschio M, Ghezzi D, Ratto GM, Cancedda L. Targeted in vivo genetic manipulation of the mouse or rat brain by in utero electroporation with a triple-electrode probe. Nat Protoc. 2016;11(3):399–412. doi: 10.1038/nprot.2016.014 26844428

[38] Lindquist RA, Guinto CD, Rodas-Rodriguez JL, Fuentealba LC, Tate MC, Rowitch DH, et al. Identification of proliferative progenitors associated with prominent postnatal growth of the pons. Nat Commun. 2016;7:11628. doi: 10.1038/ncomms11628 27188978 PMC4873968

[39] Lowenstein ED, Cui K, Hernandez-Miranda LR. Regulation of early cerebellar development. FEBS J. 2023;290(11):2786–804. doi: 10.1111/febs.16426 35262281

[40] Leto K, Arancillo M, Becker EBE, Buffo A, Chiang C, Ding B. Consensus paper: Cerebellar development. Cerebellum. 2016;15:789–828. doi: 10.1007/s12311-015-0724-2 26439486 PMC4846577

[41] Watson C, Bartholomaeus C, Puelles L. Time for radical changes in brain stem nomenclature-applying the lessons from developmental gene patterns. Front Neuroanat. 2019;13:10. doi: 10.3389/fnana.2019.00010 30809133 PMC6380082

[42] Gal JS, Morozov YM, Ayoub AE, Chatterjee M, Rakic P, Haydar TF. Molecular and morphological heterogeneity of neural precursors in the mouse neocortical proliferative zones. J Neurosci. 2006;26(3):1045–56. doi: 10.1523/JNEUROSCI.4499-05.2006 16421324 PMC3249619

[43] Comer AL, Sriram B, Yen WW, Cruz-Martín A. A pipeline using bilateral in utero electroporation to interrogate genetic influences on rodent behavior. J Vis Exp. 2020. doi: 10.3791/61350 32510510

